# Ductal tree ablation by local delivery of ethanol prevents tumor formation in an aggressive mouse model of breast cancer

**DOI:** 10.1186/s13058-019-1217-x

**Published:** 2019-11-28

**Authors:** Elizabeth Kenyon, Jennifer J. Westerhuis, Maximilian Volk, Jeremy Hix, Shatadru Chakravarty, Ethan Claucherty, Erin Zaluzec, Lisa Ramsey, Zach Madaj, Galen Hostetter, Bryn Eagleson, Erik Shapiro, Anna Moore, Lorenzo F. Sempere

**Affiliations:** 1 Precision Health Program, Michigan State University, East Lansing, MI 48824 USA; 2 Van Andel Research Institute, Grand Rapids, MI 49503 USA; 3 Department of Radiology, College of Human Medicine, Michigan State University, East Lansing, MI 48824 USA

**Keywords:** Primary prevention, Breast cancer, Prophylactic mastectomy, Mammary gland, Ductal tree, Chemical ablation, Ethanol

## Abstract

**Background:**

Prophylactic mastectomy is the most effective intervention to prevent breast cancer. However, this major surgery has life-changing consequences at the physical, emotional, psychological, and social levels. Therefore, only high-risk individuals consider this aggressive procedure, which completely removes the mammary epithelial cells from which breast cancer arises along with surrounding tissue. Here, we seek to develop a minimally invasive procedure as an alternative to prophylactic mastectomy by intraductal (ID) delivery of a cell-killing solution that locally ablates the mammary epithelial cells before they become malignant.

**Methods:**

After ID injection of a 70% ethanol-containing solution in FVB/NJ female animals, ex vivo dual stained whole-mount tissue analysis and in vivo X-ray microcomputed tomography imaging were used to visualize ductal tree filling, and histological and multiplex immunohistochemical assays were used to characterize ablative effects and quantitate the number of intact epithelial cells and stroma. After ID injection of 70% ethanol or other solutions in cancer-prone FVB-Tg-C3(1)-TAg female animals, mammary glands were palpated weekly to establish tumor latency and examined after necropsy to record tumor incidence. Statistical difference in median tumor latency and tumor incidence between experimental groups was analyzed by log-rank test and logistic mixed-effects model, respectively.

**Results:**

We report that ID injection of 70% ethanol effectively ablates the mammary epithelia with limited collateral damage to surrounding stroma and vasculature in the murine ductal tree. ID injection of 70% ethanol into the mammary glands of the C3(1)-TAg multifocal breast cancer model significantly delayed tumor formation (median latency of 150 days in the untreated control group [*n* = 25] vs. 217 days in the ethanol-treated group [*n* = 13], *p* value < 0.0001) and reduced tumor incidence (34% of glands with tumors [85 of 250] in the untreated control group vs. 7.3% of glands with tumor [7 of 95] in the ethanol-treated group, risk ratio = 4.76 [95% CI 1.89 to 11.97, *p* value < 0.0001]).

**Conclusions:**

This preclinical study demonstrates the feasibility of local ductal tree ablation as a novel strategy for primary prevention of breast cancer. Given the existing clinical uses of ethanol, ethanol-based ablation protocols could be readily implemented in first-in-human clinical trials for high-risk individuals.

## Background

An average-risk woman has a 12.5% lifelong risk of developing breast cancer [1]. For high-risk women, primary prevention strategies include surgical removal of the breasts and/or ovaries and the use of anti-estrogen medications. Despite the evidence-based effectiveness of these interventions, fewer than 30% of high-risk individuals, such as *BRCA* mutation carriers, opt for bilateral prophylactic mastectomy and fewer than 15% opt for anti-estrogen therapy as their first choice of preventative treatment [2]. The reasons for this choice are highly personal and vary among individuals and communities, but life-changing consequences and severe side effects are contributing factors [2]. For moderate- and, especially, low-risk women, there are even fewer options available to reduce their risk. Therefore, there is a need to develop new strategies for primary prevention with a focus on high-risk individuals, but strategies that can also benefit moderate- and low-risk individuals.

We seek to develop a minimally invasive procedure as an alternative to prophylactic mastectomy by intraductally delivering a cell-killing solution that locally ablates the mammary epithelial cells before they can become malignant. Our approach is informed by a growing body of literature on the use of intraductal (ID) delivery in the clinic for disease detection, such as ductography, and in preclinical and clinical research studies [3–17]. ID delivery of cytotoxic compounds, selective estrogen receptor modulators, targeted agents, and/or radioactive particles can prevent tumor formation or provide local disease control in preclinical models [4, 9–17]. The ID delivery of cytotoxic compounds (e.g., fluorouracil, pegylated liposomal doxorubicin, carboplatin) significantly reduced tumor incidence in an *N*-methyl-*N*-nitrosourea–induced rat model [4, 10, 12]. Similarly, ID delivery of pegylated liposomal doxorubicin into the mammary glands of the MMTV-Neu mouse model [10] or cisplatin to the WAP-Cre;Brca1^fl/fl^;p53^fl/fl^ mouse model [9] significantly reduced tumor incidence. Intraductal delivery of lipidoid nanoparticles containing siRNAs against Hox1A into the mammary glands of 12-week-old C3(1)-TAg transgenic mice resulted in a significant decrease in the number of tumors relative to the control group after nine weekly treatments, but tumors eventually developed in most treated glands [16]. Independent clinical studies reported a > 88% success rate in ID administration of pegylated liposomal doxorubicin or carboplatin in up to 8 ductal trees per patient [4, 5]. However, there are limitations with these ID approaches for primary prevention in humans: (i) local cytotoxic therapy (e.g., doxorubicin, fluorouracil, and cisplatin) can induce tumors in treated animals [9, 14, 18], a result that has diminished the enthusiasm for its clinical application, and (ii) local hormonal or siRNA-based therapy would require frequent and repeated intraductal injections [10, 16, 17], which makes it impractical for general clinical application.

Our approach circumvents these limitations by ablating in a single injection per ductal tree all its mammary epithelial cells rather than the targeted killing of existing, highly proliferative, pre-malignant, and/or malignant cells. In this preclinical study, we investigated a chemical ablation approach with ethanol (EtOH) as the cell-killing compound. EtOH is a readily available, stable, inexpensive, and safe compound that has long been used in the clinic. Percutaneous injection of EtOH as an ablative agent is used for the treatment of unresectable liver tumors, renal and adrenal neoplasms, pancreatic cystic tumors, and breast pseudoaneurysms [19–22] and for celiac plexus neurolysis to reduce pain [23]. Intravascular injection of EtOH as a sclerosing agent is used for the treatment of venous malformations and of spider veins and varicose veins [24–28]. Here, we demonstrate that the entire ductal tree of a mouse can be filled with a solution containing up to 70% EtOH and that such a filled ductal tree can be imaged in vivo by X-ray microcomputed tomography (microCT). Our results indicate that a 70% EtOH solution is more effective than lower concentrations at locally ablating mammary epithelial cells while causing limited collateral damage to adjacent stroma. Our prevention study using the aggressive and multifocal C3(1)-TAg mouse model of breast cancer shows that ID treatment with EtOH significantly delays tumor formation and significantly decreases tumor incidence. This preclinical study provides support for investigating local ablation as a new strategy for primary prevention of breast cancer.

## Materials and methods

### Intraductal injection procedure

FVB/NJ (*n* = 3–5 per time point and per solution; jax.org stock 001800) or FVB-Tg-C3(1)-TAg (*n* = 6–13 per solution; jax.org stock 013591) females, 9–12 weeks old, were administered 5 mg/kg of carprofen in drinking water (0.067 mg/mL of carprofen in 5% sucrose-supplemented sterile water). Then, isoflurane-anesthetized mice were injected intraductally as described [29] with either PBS or EtOH (30 to 70%) that included up to 1% of blue dye (Evans Blue injected at 0.2–0.5% [w/v]) and up to 29% of CT contrast agent (Isovue-300 injected at 87 mg iodine/mL, Bracco Diagnostics, or tantalum oxide (TaO_x_) nanocrystals (NCs) injected at 60 mM of tantalum). Detailed preparation and characterization of TaO_x_ NCs used in this study are provided in supplemental materials. Mice were injected up to 30 μL of a solution into cervical (#1, #6) or inguinal (#5, #10) glands and up to 50 μl into thoracic (#2, #3, #7, #8) or abdominal (#4, #9). All experiments were conducted under protocols approved by Institutional Animal Care and Use Committee at Van Andel Research Institute and/or Michigan State University.

### microCT imaging and analysis

Animals were serially imaged using a PerkinElmer Quantum GX microCT scanner at different times after ID injection with a solution containing Isovue-300 or Tantalum oxide nanocrystals as contrast agent. The following image acquisition scan parameters were standardized and used at each scan interval time point: 90 kVp/88 μA; field of view (FOV), 36 mm; number of slices, 512; slice thickness, 72 μm; voxel resolution, 72 μm^3^. Standard (2 min) acquisition time was used for animals used in longitudinal studies to minimize radiation exposure, and or high-resolution (4 to 14 min) acquisition time was used for short-term follow-up (less than 7 days) or terminal procedure. microCT image rendering, segmentation, and analysis of whole body or individual gland were performed using Caliper AnalyzeDirect©, v12.0 (Biomedical Imaging Resource, Mayo Clinic, Rochester, MN).

### Whole-mount dual staining and imaging

Immediately after ID injection with a solution containing Evans blue 0.2–0.5% (w/v), animals were euthanized and whole mammary glands were dissected, mounted on glass slides, and processed for carmine staining as described [30] with the following modifications. After Carnoy’s fixation, glands were dehydrated in EtOH series from 70 to 100% and incubated overnight in xylene. Cleared glands were transferred to 70% EtOH, submerged in glycerol, and scanned at 1200 dpi on an Epson Perfection V39 Photo Scanner to acquire Evans blue staining. Then, glands were rehydrated and stained with carmine alum without further modifications. Finally, glands were scanned as above to acquire carmine alum staining.

### Histological and immunohistochemical analyses

Animals were euthanized at different times after ID injections. Mammary glands were dissected and fixed in formalin for 6–8 h before processing and embedding in paraffin as previously described [31]. Four-micron sections of formalin-fixed paraffin-embedded (FFPE) tissue samples were stained with H&E and were scanned on an Aperio Versa 8 Brightfield&Fluorescence imaging system (Leica Biosystems, Buffalo Grove, IL). ImageScope tools were used for annotation and quantitative analysis. Fluorescence-based IHC multiplex assays were conducted on a Leica Bond Rx automated staining station as we described [31, 32]. These assays use covalently linked deposition of horseradish peroxidase (HRP)–reactive tyramides conjugated with fluorescent dyes (i.e., fluorescein, rhodamine, Dylight 680). The appropriate combination of primary (anti-cytokeratin 19 rat antibody at 0.576 μg/mL, Developmental Studies Hybridoma Bank, Troma-III; anti-α-smooth muscle actin rabbit antibody at 0.4 μg/mL, Abcam, ab5694; anti-vimentin chicken antibody at a 1:250 dilution, Lifespan Biosciences, Lot # 125753, LS-B291-100) and HRP-conjugated secondary antibodies (anti-rat goat/HRP at 2 μg/mL, Abcam, ab7097; anti-rabbit goat/HRP at 1 μg/mL, Biorad, 170-6515; and anti-chicken goat/HRP at 0.8 μg/mL, Santa Cruz Biotechnology, sc-2901) enabled sequential detection of multiple markers on the same tissue section, with a hydrogen peroxide blocking step in between stains to inactivate HRP from the prior round. Tissue sections were counterstained with DAPI and mounted with Prolong Gold (Invitrogen, p36930). Multi-channel images of each tissue sample were acquired and analyzed using Aperio Versa system with customized narrow-width band excitation and emission filter cubes (Chroma Technology Corp, Bellows Falls, VT).

### Statistical analysis

Unpaired Welch’s *t* tests were used to assess the statistical significance of the difference between different experimental groups of continuous values obtained from tissue analyses. Kaplan-Meier curves for tumor-free survival (the time between birth and initial time of tumor detection by palpation or time of death) were constructed separately for non-injected vs. injected glands of the same animal (experimental class) and for overall survival (the time between birth and time of death from any cause) for animals in each experimental group. For tumor-free survival data, time of death was used to censor injected glands with no evidence of tumor by palpation. Log-rank test (Mantel-Cox) was used to compare Kaplan-Meier curves between specific experimental classes or groups. GraphPad Prism 8 was used to perform these statistical analyses*.* Analyses of relative risk of tumor formation were divided into three parts comparing tumor incidence at necropsy of non-injected vs. untreated glands or injected vs. untreated glands between different experimental groups, and non-injected vs. injected glands within the same experimental group. Retrospective contingency tables of tumor incidence were constructed to determine relative risk of tumor formation between two different experimental classes and were analyzed using logistic mixed-effects models via the R v3.5.1 (https://cran.r-project.org/) package “lme4” [33] with random intercepts for each animal to account for multiplicity of tumor formation within the same animal. Tukey adjusted linear contrasts were used to test specific two-sided hypotheses of interest. We set a *p* value of 0.01 as the threshold to report statistical significance.

## Results

### Feasibility of filling the entire ductal tree with a 70% ethanol solution

Each of the 10 mammary glands of a mouse contains a single ductal tree that opens at the nipple orifice. The ductal tree elongates during pubertal development (4 to 7 weeks of age) and reaches the edge of the fat pad by 9 weeks of age. After that, growth and expansion of the ductal tree is tightly linked to the growth rate of the fat pad and of the mouse in general [34]. Terminal end buds (TEBs) are the highly proliferative structures at the tips of the elongating ductal tree that direct ductal branching. TEBs become less proliferative after puberty, and in 9-week-old young adults, they regress and become anatomically indistinguishable from terminal ducts or alveolar buds [35]. In humans, terminal ductal lobular units serve a similar function as TEBs (and later alveolar buds) and are the sites from which breast cancer predominantly arises [35, 36]. These similarities make the mouse an appropriate model to test this ID injection approach for breast cancer prevention.

We modified and implemented an ID injection procedure [29] to accommodate for the delivery of EtOH into the ductal tree(s) of female mice. This procedure places a 34-gauge needle inside the nipple opening of an isoflurane-anesthetized animal to inject the test solution. Some key improvements of our procedure include the use of gastight syringes for volatile liquids and extended anti-inflammatory treatment with 5 mg/kg of carprofen in 5% sucrose drinking water [37] from 2 days before to 7 days after ID injection in order to reduce inflammation and fibrosis associated with wound healing and tissue repair [38, 39].

**Fig. 1:**
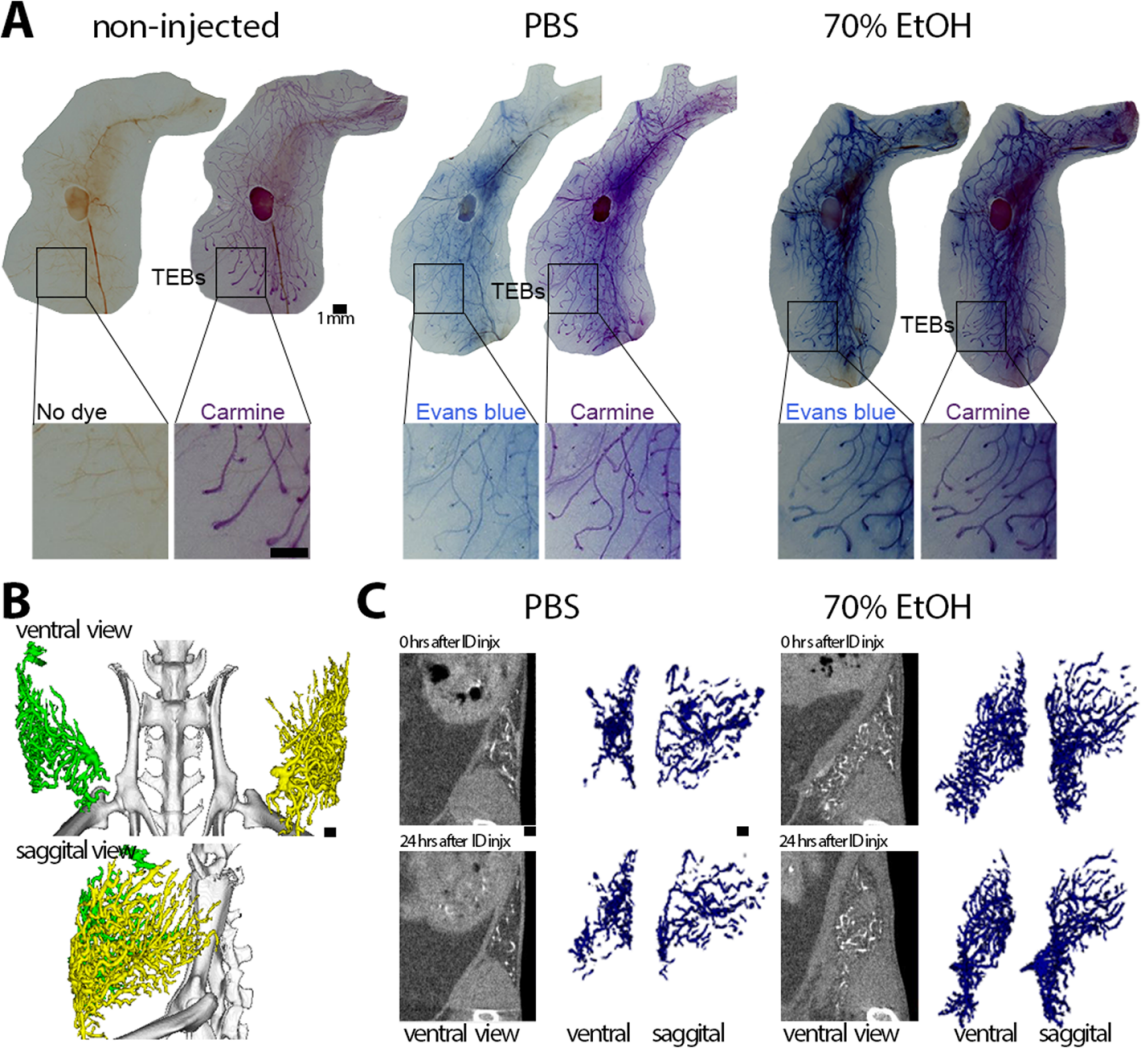
Feasibility of ductal tree filling and in vivo imaging of mammary glands with a 70% ethanol-containing solution. **a** Dual stain on whole-mount preparation of abdominal glands injected with Evans blue-containing solution of PBS or 70% EtOH. Evans blue serves to track injected solution within the lumen of the ductal tree, and carmine alum stains epithelial cells of the ductal tree. **b** Tantalum-based contrast agent-containing solution in PBS was sequentially injected within 15 min in the left abdominal (#4) and right abdominal gland (#9); 14-min high-resolution microCT scan was acquired 24 h after ID injection and processed for 3D image reconstruction. **c** Longitudinal 2-min standard microCT scans were acquired from independent animals whose abdominal glands were injected with tantalum-based contrast agent-containing solution of PBS or 70% EtOH. Different angle views and time points of the same representative glands are shown. Voxels with signal intensities from − 500 to 500 Hounsfield units in original CT slices were selected for volume rendition of diffused contrast agent. Scale bars indicate 1 mm in image panels at different magnification

Clinical uses of EtOH as an ablative or sclerosing agent consist of local delivery of tens of milliliters of 95 to 100% EtOH. In some cases, repeated administration is required due to rapid dehydration caused by EtOH. We observed this effect in initial attempts to intraductally deliver 95 to 100% EtOH: the ductal tree was occluded, preventing filling of the entire tree. To circumvent this problem, we decreased the EtOH concentration and established the feasibility of injecting a solution of up to 70% EtOH (Fig. 1). To demonstrate that 70% EtOH can fill the entire ductal tree, abdominal mammary glands were injected with PBS or 70% EtOH solution containing Evans blue dye, promptly dissected and processed for whole-mount dual staining with Evans blue and carmine alum. Overlap of Evans blue and carmine alum indicated that both PBS and 70% EtOH solution reached the terminal ends and entirely filled the murine ductal tree (Fig. 1).

### In vivo monitoring of ductal tree filling

To monitor filling of the ductal trees, we performed serial microCT imaging on animals injected with PBS or 70% EtOH solution containing iodine-based contrast agent Isovue-300 in up to three abdominal and/or thoracic mammary glands (see Additional file 1: Figure S1). Isovue-300 is used in clinical ductography [3]. In the initial image, contrast signal and rendered volumes of PBS-injected or 70% EtOH-injected were comparable; it was apparent that contrast agent-containing solution reached the ends of the ductal trees as signal was detected between the distal end of the mammary gland fat pad and the external side of the peritoneal cavity (see Additional file 1: Figure S1). There was a relatively faster loss of contrast signal in PBS-injected glands at 30 min and at later time points after ID injection compared to EtOH-injected glands (see Additional file 1: Figure S1). We surmise that this signal retention is due to the fixative effect of EtOH. However, this imaging approach does not provide sufficient resolution to identify individual ducts or branches of the ductal tree network or to quantitatively determine the volume of a filled ductal tree. To overcome these limitations, we used tantalum oxide (TaO_x_) nanocrystals (NC), a much larger contrast agent (see Additional file 1: Figure S2 and S3), with the expectation that it would have a much lower rate of outward diffusion. We performed serial microCT imaging on animals injected with PBS or 70% EtOH solution containing TaO_x_ NCs in up to three abdominal and/or thoracic mammary glands (Fig. 1). The entire ductal tree network was visualized in great detail after initial ID injection, and architectural changes could be monitored for more than 24 h thanks to local retention of TaO_x_ NCs in both PBS and EtOH solutions (Fig. 1). TaO_x_ NCs can be detected and provide a similar image reconstruction of the murine ductal tree using a standard clinical 2D/3D digital mammography system (data not shown). This in vivo imaging approach can be used to infer, but cannot directly assess at single-cell resolution, ablative effects of EtOH and stromal fibrosis.

### 70% ethanol ablates epithelial cells with limited tissue damage

**Fig. 2:**
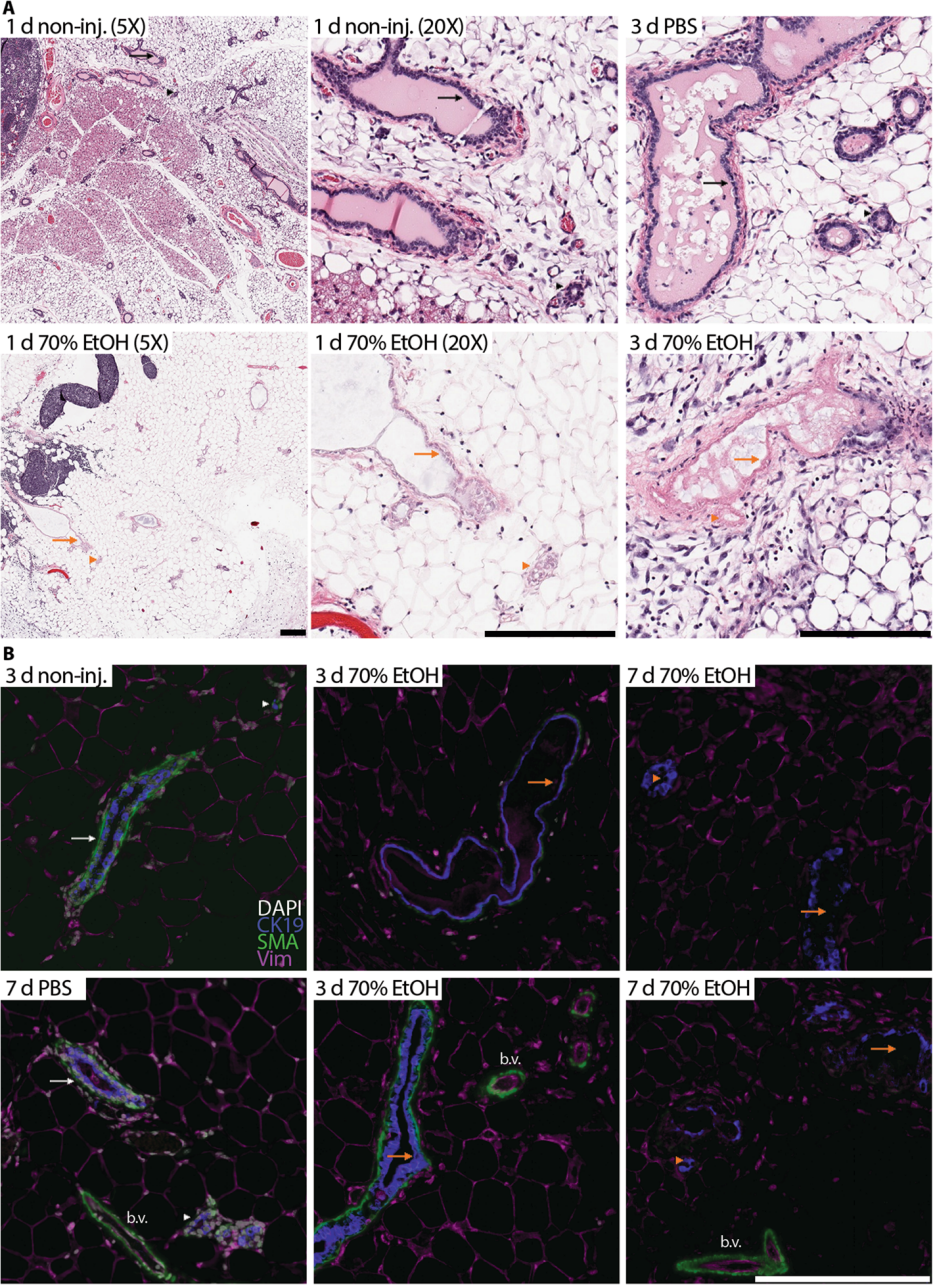
Tissue analyses of mammary glands within 7 days after ID injection of 70% ethanol. **a** Representative H&E staining of contralateral non-injected or 70% EtOH-injected glands 1 day after ID procedure (same tissues are shown at two different magnifications) and of PBS- or 70% EtOH-injected glands 3 days after ID procedure. **b** Representative multiplex staining of contralateral non-injected and PBS- or 70% EtOH-injected glands 3 days and 7 days after ID injection. After sequential detection of cytokeratin 19 (CK19), α-smooth muscle actin (SMA) and vimentin (Vim), tissues were counterstained with DAPI. Live duct (black arrow in **a** and white arrow in **b**) and ductule (black arrowhead in **a** and white arrow in **b**) or ablated duct (orange arrow) and ductule (orange arrowhead) are indicated. b.v. denotes the blood vessel with SMA-stained smooth muscle cells. Scale bars indicate 200 μm in image panels at different magnification

To obtain microscopic evidence of the extent of epithelial cell ablation and collateral tissue damage, PBS- or 70% EtOH-injected mammary glands of non-transgenic mice were dissected 1 or 3 days after the ID procedure. Contralateral non-injected mammary glands of PBS- or 70% EtOH-injected animals were also dissected as controls. Analysis of H&E-stained mammary glands revealed local killing of epithelial cells. At 1 day after the ID procedure, the number of epithelial structures in 70% EtOH-injected glands was significantly reduced, or when present, cells had hypochromatic cytoplasms and were devoid of nuclei (Fig. 2), whereas most adipocytes and blood vessels were intact. At 3 days after the ID procedure, inflammatory and fibrotic cells were observed around some of the ablated ducts and alveolar buds (Fig. 2), suggesting the start of wound healing and tissue repair. Histology of contralateral non-injected and PBS-injected mammary glands was comparable to PBS-injected glands (Fig. 2).

To complement and expand on these histological analyses, we conducted multiplex immunohistochemical analysis with cell type-specific markers to determine residual epithelial cell content and extent of peri-ductal tissue damage. Cytokeratin 19 signal was detected in “ghost” anucleate luminal epithelial cells and served as a landmark to identify and follow the clearance of ablated epithelial structures from 3 to 7 days after injection (Fig. 2). To a lesser extent, there was α-smooth muscle actin (SMA) signal in anucleate myoepithelial cells in some ablated ducts at 3 days after injection, but no SMA signal was detectable at later time points (Fig. 2). These staining patterns indicate that EtOH penetrates sufficiently from the lumen through the luminal cells to reach the myoepithelial cells. SMA signal was strong in intact, nucleated smooth muscle cells of blood vessels at all time points examined (Fig. 2). Similarly, vimentin staining highlighted the mostly intact adipocyte network (Fig. 2). These staining patterns indicate that the ablative effects of EtOH are mostly confined to the injected ducal tree(s).

### 70% ethanol provides more effective killing of epithelial cells than lower concentrations

**Fig. 3:**
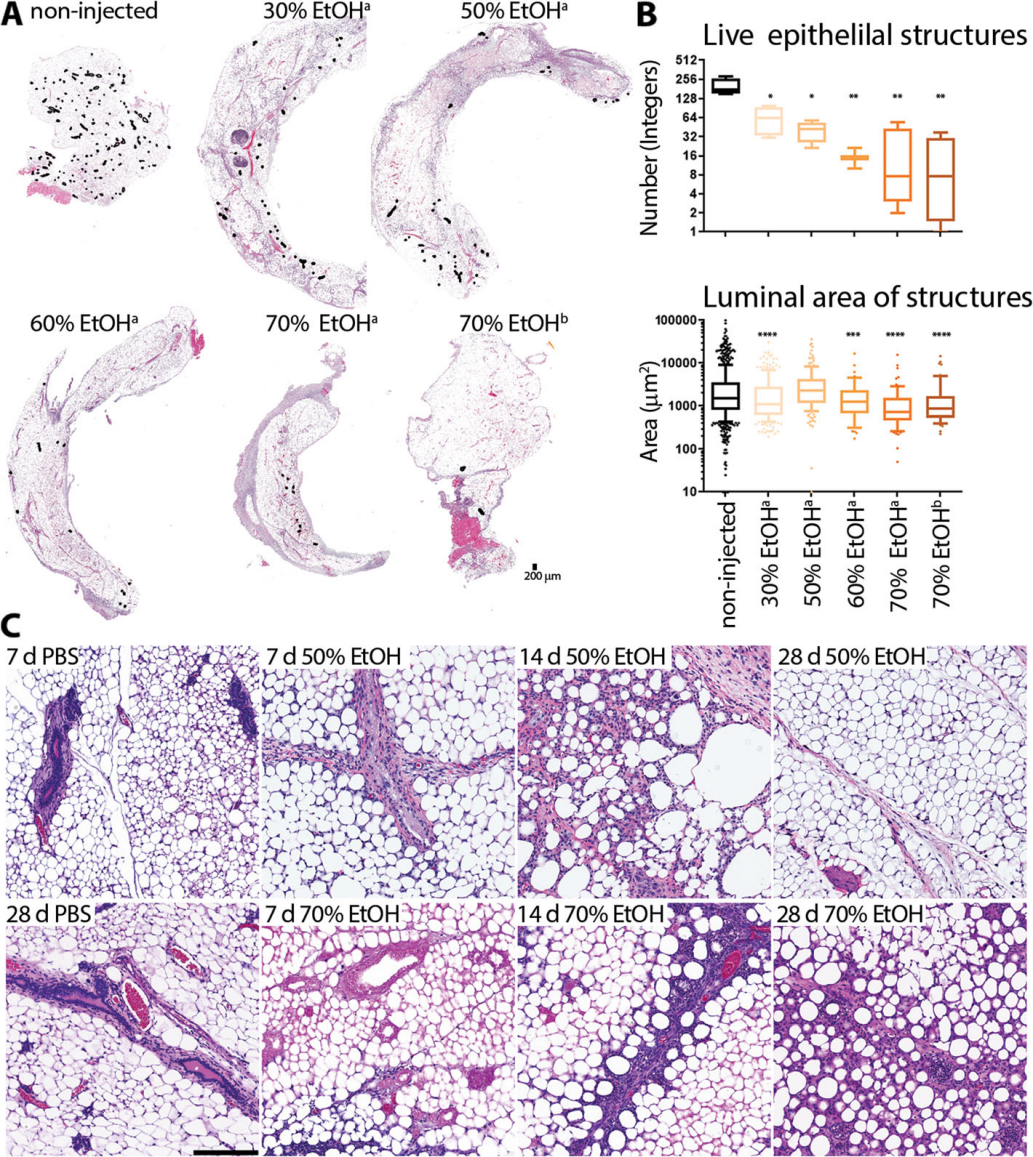
Effects of different ethanol volumes and concentration on epithelial ablation and collateral tissue damage. **a** H&E staining of representative non-injected and EtOH-injected whole mammary glands 3 days after the ID procedure. Intact, live epithelial structures are outlined in black. **b** Box-whisker plot (90/10 percentiles) of the number (in integers) of live epithelial structures or luminal areas (in μm^2^) of these structures 3 days after the injection based on whole-breast tissue cross-sectional analysis of H&E stain slides; at least four glands were quantitated per group. An epithelial structure is defined as a lobular or ductal structure with more than two adjacent live epithelial cells. Asterisks indicate *p* value of unpaired Welch’s *t* test of each EtOH-injected group compared to non-injected control animals (*0.01, **< 0.01, ***< 0.001, ****< 0.0001). **c** H&E staining of representative tissue field of 50% or 70% EtOH-injected glands at different times after ID injection

To determine whether lower concentrations of EtOH could be as effective as 70%, mammary glands were injected with different EtOH concentration (30 to 70%) and dissected 3 days after the ID procedure. The number of intact epithelial cells was significantly lower in 70% EtOH-injected glands compared to lower concentrations (30% vs. 70% EtOH, *p* < 0.001; 50% vs. 70% EtOH, *p* = 0.014) based on the interpretation of the H&E-stained tissue sections (Fig. 3). While there was not a statistically significant difference between 60% EtOH- and 70% EtOH-injected mammary glands, the estimated lower average of residual epithelial cells in this limited sample size suggested that 70% EtOH could be more effective. To evaluate collateral tissue damage and long-term effects of the ablative solution, abdominal mammary glands were injected with up to 50 μL of 50% EtOH or 70% EtOH. Collateral tissue damage was more contained and the time to complete wound healing resolution was shortened in 50% EtOH-injected glands (Fig. 3). However, maximal epithelial ablation was only consistently achieved with 70% EtOH.

### Intraductal injection of 70% ethanol prevents tumor formation in the C3(1)-TAg breast cancer model

To determine whether 70% EtOH was effective at preventing tumor formation, up to 8 mammary glands were injected per cancer-prone C3(1)-TAg female mouse with different solutions. The C3(1)-TAg is an aggressive and multifocal model of breast cancer, in which the expression of the SV40 T antigen in mammary epithelial cells is driven by the rat prostatic steroid binding protein C3(1) promoter [40]. Animals received the ID injections between 9 and 12 weeks of age when there were still no hyperplastic or in situ carcinoma lesions [16, 40]. The following experimental and control groups were used in this prevention study. There were three experimental groups that received ID injections of different EtOH concentrations and/or different formulations. One group received injections of 50% EtOH in PBS and iodine-containing contrast agent. This group served mainly to determine if partial ablation would still provide protection or would accelerate tumor formation due to epithelial cell regeneration in an inflammatory milieu. The other two experimental groups received ID injections of 70% EtOH either in deionized distilled water or in a solution of PBS and iodine-containing contrast agent to compare if diluent affected ablative properties. Control groups included a group that received no treatment, a non-injected control group that received anti-inflammatory treatment but no ID injections, and a PBS-injected group that received injections in some mammary glands. These control groups served to separate the effects of anti-inflammatory treatment and the ID procedure.

**Fig. 4:**
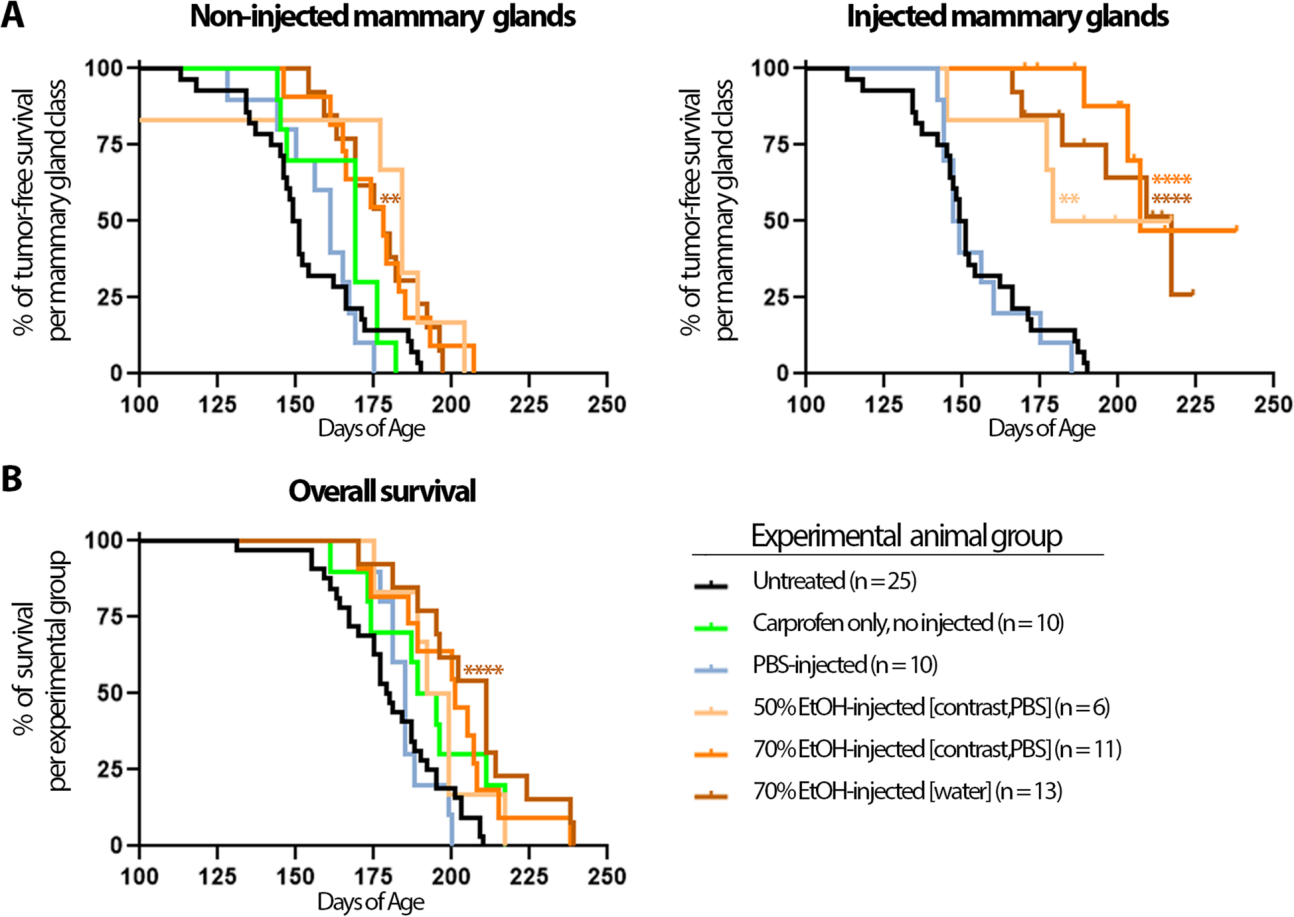
Kaplan-Meier curves of tumor-free and overall survival. **a** Kaplan-Meier curves for tumor-free survival were plotted separately for non-injected and injected glands from the same animal, where appropriate. **b** Kaplan-Meier curves for overall survival were plotted for all experimental groups. Legend indicates which plotted curve corresponds to each experimental group and the number of animals per group (**a**, **b**). Colored asterisks indicate *p* value of log-rank test of matched experimental class or group compared to non-injected control animals (**< 0.01, ****< 0.0001); see Tables 1 and 2 for more details

The two primary endpoints for this study were tumor latency and tumor incidence. A secondary endpoint was overall survival, which was reached in all cases by tumor burden and not by cancer-related death. Animals were palpated weekly from 14 weeks of age until they met euthanasia criteria. The first instance of tumor formation and number of tumors at necropsy were recorded separately for non-injected and injected mammary glands per mouse, where appropriate (Fig. 4). ID injections of EtOH significantly delayed tumor formation in EtOH-injected mammary glands in all experimental groups (50% or 70% in PBS/contrast, 70% in water) compared to the untreated group (Fig. 4 and Table 1). ID injection of EtOH also delayed tumor formation in non-injected mammary glands in all experimental groups, though only reached statistical significance in the 70% EtOH-in-water group (Table 1). We noted, but did not investigate further whether this is an abscopal effect of injected glands systemically affecting the non-injected glands or simply a reduction in the number of mammary glands susceptible to develop tumors. In either case, the preventative effect of EtOH injection was significantly higher in injected glands than in non-injected glands in both 70% EtOH-injected experimental groups based on delayed tumor latency (Table 1). Overall survival of 70% EtOH-injected animals in both experimental groups was significantly longer compared to the untreated control group (Fig. 4 and Table 2). Similarly, there was a significant reduction in the risk of tumor formation in 70% EtOH-injected glands compared to the untreated control group or non-injected glands within the 70% EtOH-injected experimental groups (Table 3). Intriguingly, both the injected and non-injected mammary glands in the PBS-injected control group had a higher tumor incidence than the untreated and non-injected control groups, suggesting that non-ablative ID injection may create a pro-tumorigenic environment that negates protection of anti-inflammatory treatment (Table 3). Together, these results indicate that 70% EtOH in either of the tested formulations is effective, and more so than 50% EtOH, at preventing tumor formation in the C3(1)-TAg breast cancer model.

**Table 1:** Intraductal injection of ethanol delays onset of tumor formation

		Non-injected vs. non-injected glands			Non-injected vs. injected glands		
Animal groups	Animals (*n*)	Latency (days)	Glands (*n*)	*p* value	Latency (days)	Glands (*n*)	*p* value
Untreated vs. not injected	25 vs. 10	150 vs. 169	250 vs. 100	0.4279	n/a	n/a	n/a
Untreated vs. PBS-injected	25 vs. 10	150 vs. 161	250 vs. 43	0.9110	150 vs. 148	250 vs. 57	0.6570
Untreated vs. 50% EtOH-injected^a^	25 vs. 6	150 vs. 184	250 vs. 36	0.0366	150 vs. 198	250 vs. 24	0.0049
Untreated vs. 70% EtOH-injected^a^	25 vs. 11	150 vs. 178	250 vs. 58	0.0151	150 vs. 207	250 vs. 52	< 0.0001
Untreated vs. 70% EtOH-injected^b^	25 vs. 13	150 vs. 176.5	250 vs. 35	0.0059	150 vs. 217	250 vs. 95	< 0.0001
Within PBS-injected	10	n/a	n/a	n/a	161 vs. 148	43 vs. 57	0.7274
Within 50% EtOH-injected^a^	6	n/a	n/a	n/a	184 vs. 198	36 vs. 24	0.2987
Within 70% EtOH-injected^a^	11	n/a	n/a	n/a	178 vs. 207	58 vs. 52	0.0002
Within 70% EtOH-injected^b^	13	n/a	n/a	n/a	176.5 vs. 217	35 vs. 95	0.0006

Median latency in days of age is provided for indicated animal treatment group or mammary gland treatment class. *p* values were calculated by the log-rank test (Mantel-Cox) comparing tumor-free survival between indicated treatment groups or classes (reference vs. test). ^a^EtOH was diluted in sterile PBS with iodine-containing contrast agent. ^b^EtOH was diluted in sterile water

**Table 2:** Intraductal injection of ethanol increases overall survival

Group comparison	Animals (*n*)	Overall survival (days)	*p* value
Untreated vs. not injected	25 vs. 10	179.5 vs. 192	0.0394
Untreated vs. PBS-injected	25 vs. 10	179.5 vs. 185	0.9655
Untreated vs. 50% EtOH-injected^a^	25 vs. 6	179.5 vs. 195.5	0.1199
Untreated vs. 70% EtOH-injected^a^	25 vs. 11	179.5 vs. 201	0.0184
Untreated vs. 70% EtOH-injected^b^	25 vs. 13	179.5 vs. 211	< 0.0001
50% EtOH-injected^a^ vs. 70% EtOH-injected^a^	6 vs. 11	195.5 vs. 201	0.5495
50% EtOH-injected^a^ vs. 70% EtOH-injected^b^	6 vs. 13	195.5 vs. 211	0.1801
70% EtOH-injected^a^ vs. 70% EtOH-injected^b^	11 vs. 13	201 vs. 211	0.2789

Median overall survival in days of age is provided for indicated animal treatment groups. *p* values were calculated by the log-rank test (Mantel-Cox) comparing overall survival between indicated treatment groups (reference vs. test). ^a^EtOH was diluted in sterile PBS with iodine-containing contrast agent. ^b^EtOH was diluted in sterile water

**Table 3:** Intraductal injection of ethanol reduces the risk of developing cancer in a C3(1)-TAg mouse model (tumor incidence at necropsy)

	Non-injected vs. non-injected glands			Non-injected vs. injected glands		
Animal groups	Tumor incidence (%, *n*)	RR (95% CI)	*p* value	Tumor incidence (%, *n*)	RR (95% CI)	*p* value
Untreated vs. not injected	34% (85/250) vs. 28% (28/100)	1.21 (0.76–1.93)	0.6848	n/a	n/a	n/a
Untreated vs. PBS-injected	34% (85/250) vs. 58% (25/43)	0.58 (0.39–0.86)	0.0147	34% (85/250) vs. 52% (30/57)	0.64 (0.44–0.94)	0.0347
Untreated vs. 50% EtOH-injected^a^	34% (85/250) vs. 36% (13/36)	0.94 (0.51–1.72)	0.9924	34% (85/250) vs. 16% (4/24)	2.12 (0.67–6.70)	0.2328
Untreated vs. 70% EtOH-injected^a^	34% (85/250) vs. 51% (30/58)	0.65 (0.44–0.97)	0.0555	34% (85/250) vs. 9.6% (5/52)	3.53 (1.21–10.29)	0.0048
Untreated vs. 70% EtOH-injected^b^	34% (85/250) vs. 57% (20/35)	0.61 (0.39–0.94)	0.0599	34% (85/250) vs. 7.3% (7/95)	4.76 (1.89–11.97)	< 0.00001
Within PBS-injected	n/a	n/a	n/a	58% (25/43) vs. 52% (30/57)	1.10 (0.77–1.57)	0.6765
Within 50% EtOH-injected^a^	n/a	n/a	n/a	36% (13/36) vs. 16% (4/24)	2.25 (0.83–6.13)	0.0579
Within 70% EtOH-injected^a^	n/a	n/a	n/a	51% (30/58) vs. 9.6% (5/52)	5.37 (2.24–12.86)	< 0.00001
Within 70% EtOH-injected^b^	n/a	n/a	n/a	57% (20/35) vs. 7.3% (7/95)	7.77 (3.58–16.85)	< 0.00001

Tumor incidence indicates the number of glands with tumors at necropsy in each gland class (i.e., untreated, non-injection, or injected). Relative risk (RR) indicates the likelihood of having a tumor in one class of mammary glands vs. another within a treatment group (same animals) or among treatment groups (independent animals) with 95% confidence interval (CI). RR > 1 indicates a lower risk of tumor formation for that particular group or intervention compared to the reference group (reference vs. test). Logistic mixed-effects models with random intercepts for each animal and Tukey-adjusted linear contrasts were used for this per-gland analysis to account for multiplicity of tumor formation in the same animal. ^a^EtOH was diluted in sterile PBS with iodine-containing contrast agent. ^b^EtOH was diluted in sterile water

### No iatrogenic cancer or other long-term adverse effects after ID injection of ethanol

**Fig. 5:**
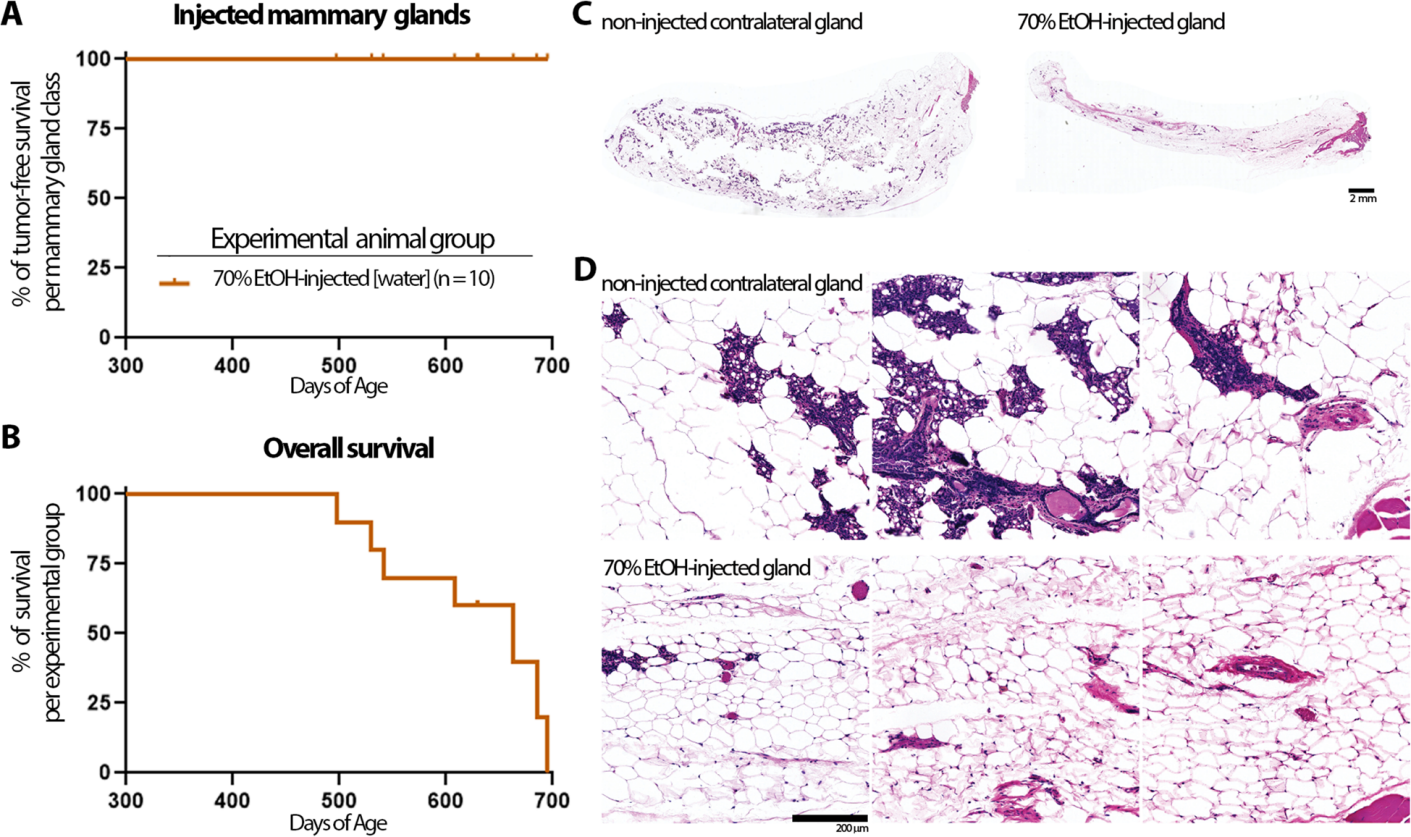
No iatrogenic breast cancer in aged mice after ID EtOH injection. **a**, **b** Kaplan-Meier curve for tumor-free survival (**a**) and for overall survival (**b**) was plotted for non-transgenic FVB animals injected with 70% EtOH in multiple glands. All animals died of natural causes, with the exception of three animals that were euthanized at designated time point for tissue analyses and censored at this time (630 days of age) for tumor-free and overall survival curves. **c**, **d** Representative H&E staining of contralateral non-injected or 70% EtOH-injected glands 18 months after ID procedure. Whole gland tissue is shown in **c** and representative field along each gland is shown at high power magnification in **d**. **d** Some residual epithelial structures can be observed only in the upper left quadrant of the left image of the EtOH-injected gland. Size of the scale bar is 2 mm for images in **c** and 200 μm for images in **d**

To evaluate longitudinally any potential side effects or complications of this ID ablation procedure such as infection, open wounds, or chronic inflammation, non-transgenic animals were injected in up to 6 mammary glands with up to 50 μL of 70% EtOH. Animals were monitored daily until the designated time point for mammary gland tissue collection. No signs of infection or open wounds nor any changes in grooming, locomotive, or social behavior that would indicate pain or discomfort were noted. Touch test with von Frey filaments before or after ID injection did not detect any withdrawal response in either non-injected or injected glands at target forces that elicit a response in the rear paw (data not shown). Ten animals were followed for more than 14 months after 70% EtOH injection in multiple mammary glands. Three of these animals were selected for tissue collection at 18 months after ID injection in order to perform tissue analyses (Fig. 5). The other seven animals died of natural causes with no signs of breast cancer or other diseases with a median overall survival of > 630 days (Fig. 5).

## Discussion

We investigated and recorded short-term and long-term effects of ID 70% EtOH injections on breast biology and mouse physiology and evaluated whether any of these effects and/or experimental requirements of EtOH-based ablative procedure pose an obvious impediment for human translation. The short-term effects of ID EtOH injection were relatively mild when precautions were taken. The two short-term side effects of EtOH injection were ethylic intoxication and skin laceration. Mice injected with 150 μL of 70% EtOH (3 mammary glands) exhibited signs of alcohol intoxication (about 0.4 g/dL of EtOH content in blood) which was minimized by intraperitoneal injection of 5% sucrose in PBS before and after the ID procedure; animals fully recovered within 4 h after the ID procedure. For the injection of more than 3 glands per mouse, a sequential procedure was performed to allow enough recovery time. The risk of alcohol intoxication in women will be much lower; injection of ductal trees in both breasts, assuming 24 main ducts [41, 42] and 2 mL per duct [3, 8], with 70% EtOH will result in less than 0.1 g/dL of EtOH content in blood, which approximates to drinking three glasses of wine (15 oz), and may cause mild impairment. The total volume of 50 mL of 70% EtOH and total EtOH quantity (27.61 g) is comparable to the up to 50 mL of 95–100% EtOH (up to 39.45 g) reported in percutaneous EtOH injection procedures for the treatment of liver tumors [20] and venous malformations [28]. Skin lacerations were observed in some mice due to small leakage of EtOH from the injected duct or from the needle while exiting the nipple. These were topical lacerations due to direct skin contact and not from internal tissue damage. The risk of these lacerations will be much lower by taping the nipple after ductal injection as it is routine practice for ductography [3, 8]. We did not observe any long-term effects of EtOH injection; specifically, none of the mice had open wounds or infection in injected glands nor did they exhibit any overt signs of pain, distress, or discomfort. Histologically, glands injected with 50 μL of 70% EtOH started to heal by 14 days after the injection. We observed that by 1 month there was limited scarring and subsiding inflammation (Fig. 3). Long-term follow-up of glands showed no signs of scarring or inflammation at 18 months after EtOH injection (Fig. 5). Nonetheless, improvement in the EtOH formulation such as addition of ethyl cellulose as gelling agent to limit the outward diffusion of EtOH should be considered to further minimize the collateral tissue damage. The use of ethyl cellulose for this purpose is routine in some sclerosing protocols for the treatment of venous malformation [26, 27] and has also been reported to improve ablative efficacy in percutaneous EtOH injection in a preclinical model of liver cancer [43].

The safety bar for new preventive agents and approaches is very high. Thus, safety is our primary concern when considering translation of this ID procedure. While the International Agency for Research on Cancer considers EtOH in alcoholic beverages to be carcinogenic to humans (https://monographs.iarc.fr/wp-content/uploads/2018/06/mono100E-11.pdf), this conclusion is based on chronic exposure to EtOH. The exact molecular mechanism(s) of how EtOH increases cancer risk have not been completely established; EtOH metabolization into acetaldehyde, a toxic chemical that can cause DNA damage and DNA-protein crosslinking, is considered a main contributing mechanism for EtOH-induced cancer (https://monographs.iarc.fr/wp-content/uploads/2018/06/mono100E-11.pdf). Nonetheless, we are not aware of any reports of iatrogenic cancer linked to clinical uses of EtOH. Acute exposure to EtOH in mice does not cause significant DNA damage [44]. In addition, we have no evidence from our study that EtOH injection promotes or enhances tumor initiation in cancer-prone C3(1)-TAg animals followed up until they met euthanasia criteria (Fig. 4) nor in non-transgenic animals followed up more than 15 months after ID EtOH injection (Fig. 5). Note that breast tumors were observed in non-transgenic mice within a year of exposure to ID chemotherapy or immunoradiotherapy [9, 13, 14, 18].

We need to mention several limitations of our study. While we attempted to inject as many mammary glands as technically possible, most mice were injected in fewer than 6 glands. Thoracic and abdominal glands were more often injected than cervical or inguinal glands (see Additional file 1: Table S1). Other studies showed the feasibility of injecting all 10 glands [10, 16], but in our hands, cervical glands are not often suitable for injection. There is no need to inject all glands to assess whether EtOH can prevent breast cancer in injected glands with statistical methods that we and other groups used [4, 9, 10]. However, glands that were more accessible for injection may not develop tumors at the same rate as the non-injected glands (see Additional file 1: Figure S1; see also reference [45]). We addressed in part this potential bias with a control group of animals injected with PBS in a similar number of glands and locations (see Additional file 1: Figure S1). In these animals, tumors arose with similar latency and tumor incidence in injected and non-injected glands. Tumor latency in 70% EtOH-injected glands was significantly delayed and tumor incidence significantly reduced compared to PBS-injected glands. Intriguingly, tumor incidence was significantly increased in PBS-injected animals, but not tumor latency, compared to untreated control animals (Table 1). Thus, we used the untreated control group as baseline reference for statistical analysis rather than the PBS-injected group. Typically, the tumor burden in non-injected glands was the reason for euthanasia in EtOH-injected animals, which limited the follow-up time in EtOH-injected glands that might have eventually developed breast tumors. Future prevention studies in other mouse models such as genetically engineered MMTV-Neu [10] and WAP-Cre;Brca1^fl/fl^;p53^fl/fl^ [9] with longer latency and/or lower multiplicity and/or inducible models by ID injection of Cre recombinase [46] would be useful to provide an extended follow-up period and determine the general application of the EtOH ablation procedure to tumor formation driven by different molecular alterations and cell of origin. There are technical challenges in consistently and reproducibly achieving ablation of all mammary epithelial cells of an injected ductal tree. The ductal tree is not completely hollow; it may contain proteinaceous secretions and cellular debris that could affect accessibility and diffusion and/or dilute the EtOH concentration. Architectural changes during the estrous cycle may also affect filling of the ductal tree. These changes are more prominent during alveolar growth in diestrus and subsequent alveolar collapse in proestrus [47]. Thus, injection of the same EtOH volume in ductal tree of different mammary glands of the same mouse or in different mice may have not caused the same rate of ablation providing at least partial explanation as to why tumors still developed in EtOH-injected mammary glands. We observed a varying degree of residual epithelial cell structures in some of the injected glands in non-transgenic animals (Figs. 3 and 5). By design, we injected up to 50 μL of ablative solution, and it is likely that some ductal trees were not fully filled and a larger volume could have effectively ablated more or all of the cells. Due to the size and fragility of the mouse nipple, cannulation to deliver a contrast solution to determine the exact volume requirements before filling with the EtOH solution would be extremely challenging. However, these volume measurements and architectural difference per individual ductal tree could be obtained in women by combining a standard ductography procedure with a preparatory solution for flushing each ductal tree as is often done for ductal lavage collection.

We wish to acknowledge that previous preventive studies directly investigated the idea of epithelial cell ablation as treatment or observed epithelial cell ablation as an indirect consequence of treatment. In a chemically induced rat model, ID injection of a suicidal gene adenoviral vector with the intent of ablating proliferating cells of the TEB to prevent tumor formation paradoxically promoted tumor initiation and increased tumor incidence [11]. Despite the high efficiency transduction, high expression of thymidine kinase, and 50–90% ablation rate upon suicidal gene activation by ganciclovir administration, intraductally treated rats developed tumors at shorter latency than control rats exposed to *N*-methyl-*N*-nitrosourea [11]. In genetically engineered mouse models, ID treatment with doxorubicin or cisplatin caused partial destruction of the ductal tree and cell ablation [4, 9], but these treatments were not more effective than those that did not cause cell ablation [4].

Given the existing clinical uses of EtOH and relatively straightforward ID injection procedure, EtOH-based ablation protocols could be readily implemented in clinical trials for primary prevention of breast cancer. We envision that this ablative procedure would most closely approximate the cosmetic treatment of venous malformations. Our preclinical procedure is in line with clinical sclerosing therapy for venous malformation in which patients receive treatment under systemic anesthesia followed by 2 days of anti-inflammatory medications such as NSAIDs that may be extended for a few more days to reduce local inflammation and any possible pain [26]. Typically, the sclerosing therapy reduces chronic pain associated with swollen and deformed vasculature in most patients; this intervention can cause short-term pain in a few patients that is easily managed with medication. In contrast, the physiological response to mastectomy includes local and systemic inflammation, and the scale of these responses and the pain management plan depend on the surgical procedure, including musculoskeletal manipulation and tissue advancement for reconstruction. Typical peri-operative pain management includes a combination of analgesics with regional anesthesia, narcotics, benzodiazepines, and anti-inflammatory medications such as NSAIDs over a course of 2–6 weeks. Regrettably, at least 25% of patients suffer from chronic pain or post-mastectomy chronic pain syndrome [48, 49].

We foresee challenges for clinical implementation that will need to be addressed. (i) The EtOH concentration may need to be adjusted for application in humans due to anatomical differences from rodents, such as the size and complexity of their ductal trees; progressive scalability studies in rats [10, 17] or rabbits [50], and larger animal models, such as pigs, will be required before translation to humans. (ii) If the total amount of EtOH is much larger than 50 mL due individual ducts accommodating more than estimated 2 mL as reported for intraductal delivery of chemotherapeutic agents [4, 5], sequential treatment of each breast in separate visits and/or intravenous administration of thiamine and sugar solution may be required to minimize effects of EtOH intoxication. (iii) The typical human breast is composed of 8–12 ductal trees [41, 42]; successful cannulation and injection in each main duct will be needed to preventively treat the whole breast. (iv) Evidence that the entire tree was filled will be required. EtOH could be injected with a radiocontrast agent to visualize ductal tree filling (as shown in Fig. 1 for mice) using existing ductography methods [3, 8] or could be intrinsically labeled for visualization using other imaging modalities such as EtOH-^17^O for magnetic resonance imaging [51]. In some cases, hyperplastic or proliferative regeneration may occlude a duct, preventing filling of the entire ductal tree. It may be possible to dilate or clear passage of such a duct by flushing with a preparatory solution. (v) Pathological evidence that all epithelial cells were ablated may be required. This would be feasible in first-in-human trials in women undergoing elective prophylactic mastectomy, from whom tissue samples of the entire breast will be accessible. We would follow a similar experimental design as described for ID administration of chemotherapeutic agents [4, 5]. Going forward, cell ablation would need to be assessed using in vivo imaging and/or limited tissue sampling by core needle biopsy, since pathological assessment of whole-breast tissue would defeat the purpose of this local, minimally invasive procedure. (vi) The amount of pain, scarring, and other complications associated with this procedure will need to be determined and compared to those of a mastectomy. This procedure will need to have a good safety profile if it were to replace mastectomy in a preventive setting. As with mastectomy, this procedure will eliminate the ability of a woman to lactate or breastfeed.

## Conclusions

This preclinical study provides support for further investigating local ablation as a new strategy for the primary prevention of breast cancer in high-risk individuals. This study could also stimulate the evaluation of other chemical and/or thermal ablation strategies. This ID procedure could lead to a breakthrough in breast cancer prevention by providing a universal prophylactic intervention, one not only for high-risk individuals but potentially for those with moderate or low risk; by improving the individual’s quality of cancer-free life; and by decreasing the personal and societal costs of breast cancer.

## Supplementary information

**Additional file 1. **This pdf file includes a supplementary table and three supplementary figures. **Figure S1.** In vivo imaging of mammary glands with Isovue-300 in 70% ethanol-containing solution. **Figure S2.** Characterization of tantalum oxide nanocrystals by transmission electron microscopy. **Figure S3.** Characterization of tantalum oxide nanocrystals by dynamic light scattering. **Table S1.** Tumor formation in non-injected and injected mammary glands assessed at necropsy.

## Data Availability

All data generated or analyzed during this study are included in this manuscript. Further details are available on request.

## Abbreviations

CK19: Cytokeratin 19
EtOH: Ethanol
FFPE: Formalin-fixed paraffin-embedded
HRP: Horseradish peroxidase
ID: Intraductal
microCT: X-ray microcomputed tomography
NC: Nanocrystal
SMA: α-Smooth muscle actin
TaO_x_: Tantalum oxide
TEB: Terminal end bud
Vim: Vimentin
