# Supplementary material for: Ductal tree ablation by local delivery of ethanol prevents tumor formation in an aggressive mouse model of breast cancer
Source: Breast Cancer Res. 2019 Nov 28;21:129. doi: 10.1186/s13058-019-1217-x (PMC6883550; doi:10.1186/s13058-019-1217-x)
Supplement: Supplementary file 1 — Additional file 1. This pdf file includes a supplementary table and three supplementary figures. Figure S1. In vivo imaging of mammary glands with Isovue-300 in 70% ethanol-containing solution. Figure S2. Characterization of tantalum oxide nanocrystals by transmission electron microscopy. Figure S3. Characterization of tantalum oxide nanocrystals by dynamic light scattering. Table S1. Tumor formation in non-injected and injected mammary glands assessed at necropsy. [file 13058_2019_1217_MOESM1_ESM.pdf]

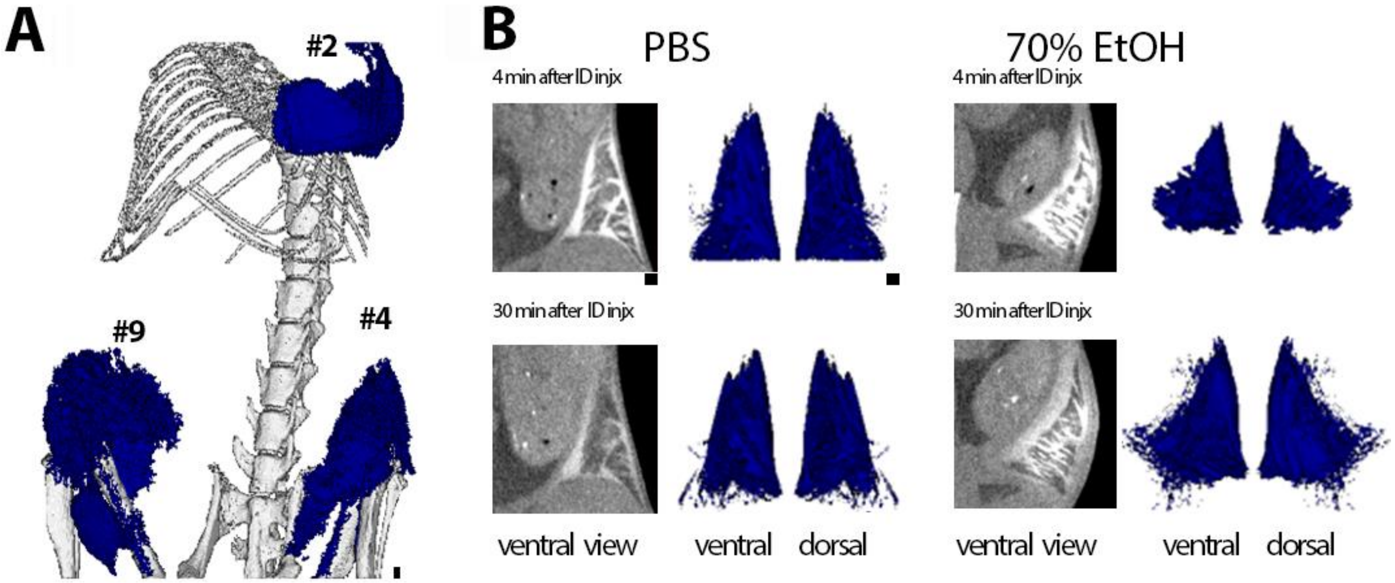

Supplementary Figure 1. *In vivo* imaging of mammary glands with Isovue-300 in 70% ethanol-containing solution. A) Iodine-based contrast agent-containing solution of 70% EtOH was sequentially injected within 15 min in left abdominal (#4), left thoracic (#2), and right abdominal gland (#9); full-body microCT scan was acquired immediately after the last ID injection. **B)** Sequential 4 min. high-resolution microCT scans were acquired from independent animals whose abdominal glands were injected with iodine-based contrast agent-containing solution of PBS or 70% EtOH. Different angle views and time points of the same representative glands are shown. Voxels with signal intensities from -500 to 500 Hounsfield units in original CT slices were selected for volume rendition of diffused contrast agent. Scale bars indicate 1 mm in image panels at different magnification.

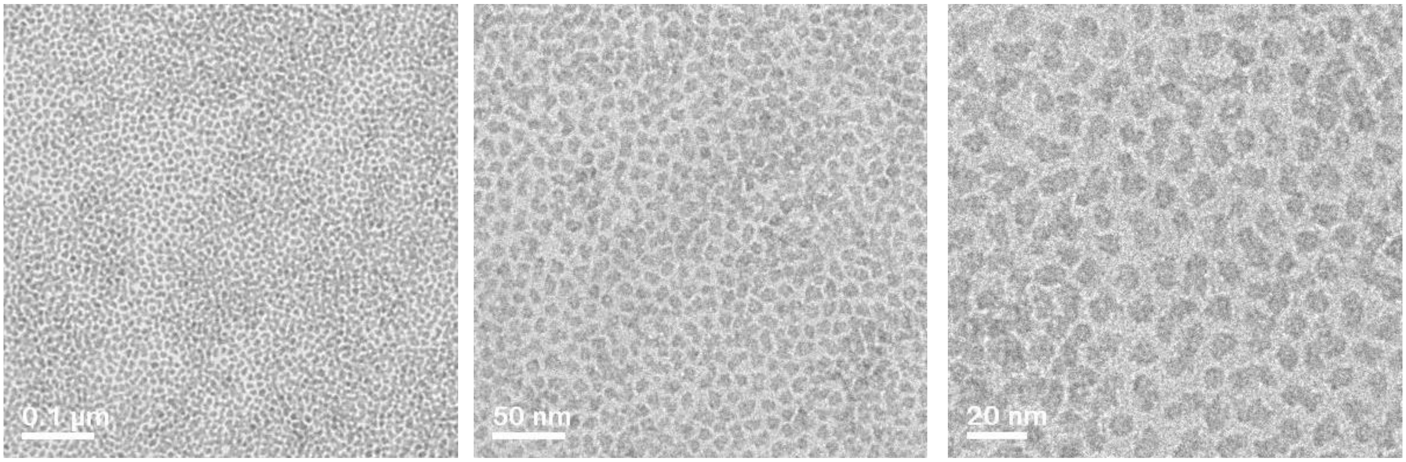

Supplementary Figure 2. Characterization of Tantalum oxide (TaO_x) nanocrystals (NCs) by transmission electron microscopy (TEM). TEM image show excellent homogeneity in size and morphology of TaO_x NCs. TEM images were acquired on a 2200FS JEOL electron microscope. These TaO_x NCs were prepared as follows: In a 250 mL, one neck round bottom flask, fitted with a septa, IGEPAL®-CO-520 (average M_n 441, ALDRICH, 23.0 g), Cyclohexane (≥99%, A.C.S. spectrophotometric grade, SIGMA-ALDRICH, 200 mL) and Ethanol (200 Proof, Anhydrous, KOPTEC USP, 2.5 mL), were added and the contents were stirred to obtain a clear solution. To this stirring mixture, a solution of Sodium Hydroxide (100 mM, 2.5 mL) was added and the micro-emulsion was sonicated in a water bath to ensure homogeneity. Next, Tantalum (V) ethoxide, (Ta_2O_5, 99.98% trace metal basis, ALDRICH, 0.5 mL) was added in one portion and the contents were stirred at ambient temperature for 20 minutes. To the micro-emulsion mixture containing uncoated TaOx NCs, 2-[Methoxy (polyethyleneoxy)-9-12-propyl]trimethoxysilane (PEG-Silane, tech-90, MW 591-723, GELEST INC., 3.0 mL), quickly followed by (3-Aminopropyl)trimethoxysilane (APTMS, 97%, ALDRICH, 0.028 mL) were added. The resulting milky white suspension solution was stirred at room temperature for 16 h. After 16 h, the reaction mixture was diluted to three times volume using a 1:1 mixture of Ethyl Ether (Anhydrous, Certified ACS, Fisher Scientific, 110 mL) and Hexane (meets ACS specifications, VWR Chemicals, 110 mL) and the NCs were isolated via centrifugation (15,000 rpm, 10 minutes, 10 °C) as white oily residue. This residue was suspended in ethyl ether and washed using a similar centrifugation procedure twice. The supernatants were discarded and the residue pellet so obtained was suspended in 100 mL Ethanol and Methoxy-poly(ethylene-glycol)-succinimidyl glutarate (m-PEG-SG-2000, Average MW 2000, LAYSAN BIO INC., 50 mg) was added to it. The contents so obtained were stirred at room temperature in the dark for 12 h. Then, the solvent was removed on a rotary evaporator to reduce the volume to about 5 mL. This final residual solution was dissolved in water (10 mL) and transferred to Dialysis Membrane bags (SPECTRA/POR® 6 Dialysis Membrane, Standard RC Tubing, MWCO: 1 kD), clipped at both ends and dialyzed against water with regular change of external media after 2, 4, 16, 4, 4 and 16 h. After extensive dialysis, the contents in the dialysis bags were lyophilized to obtain the TaO_x NCs as a white fluffy powder. Product Yield: 940 mg. Ta% = 30% (calculated from ICP-OES).

Results

			Size (d.nm):	% Number	Width (d.nm...
Z-Average (d.nm):	43.05	Peak 1:	9.036	100.0	2.616
PdI:	0.458	Peak 2:	0.000	0.0	0.000
Intercept:	0.758	Peak 3:	0.000	0.0	0.000

Result quality : Good

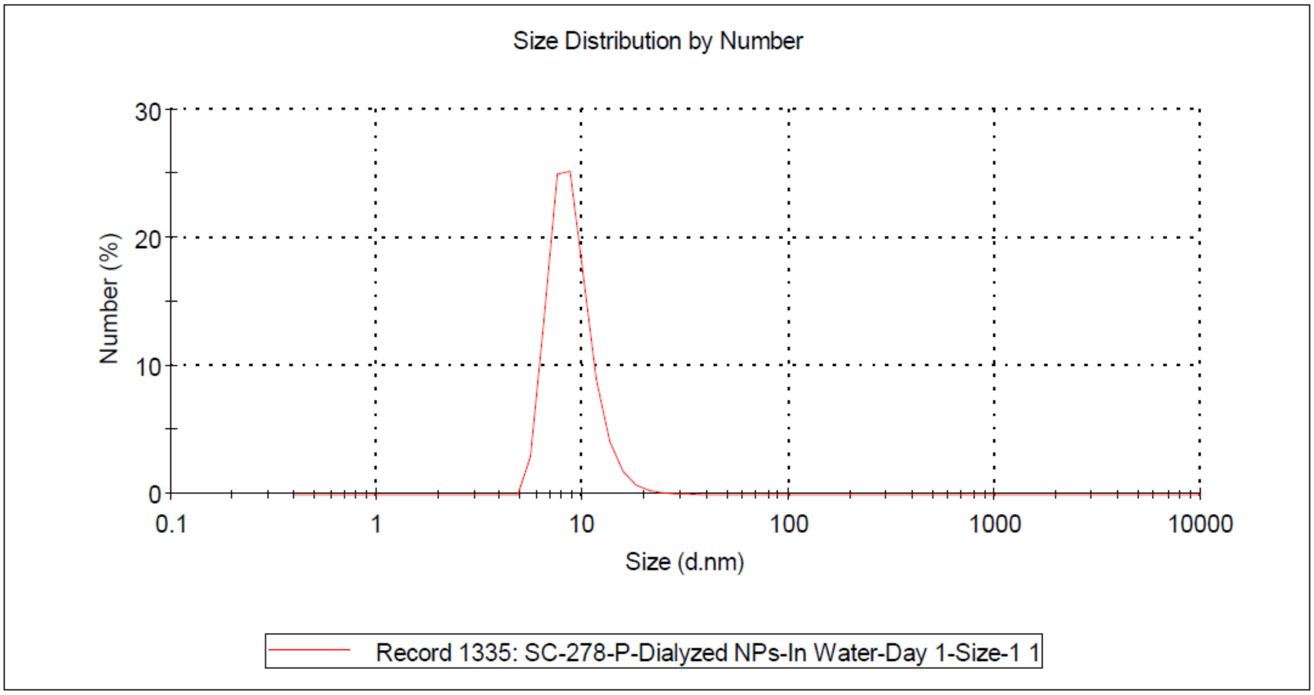

Supplementary Figure 3. Characterization of TaO_x nanocrystals by Dynamic Light Scattering (DLS). DLS plot shows a narrow range of particle size distribution. TaO_x were analyzed in hydrophilic conditions by dissolving TaOx nanocrystals at 1 mg/mL in water. DLS plot was acquired on a Zetasizer instrument (Malvern, USA).

		Tumor in non-injected gland										Tumor in injected gland									
**ID**	**experimental group**	**1**	**2**	**3**	**4**	**5**	**6**	**7**	**8**	**9**	**10**	**1**	**2**	**3**	**4**	**5**	**6**	**7**	**8**	**9**	**10**
90	untreated	N	N	N	N	N	Y	N	N	N	N										
101	untreated	Y	Y	N	N	N	Y	N	Y	Y	Y										
106	untreated	Y	Y	N	N	N	N	N	Y	N	Y										
108	untreated	Y	Y	N	Y	Y	Y	N	N	N	N										
109	untreated	Y	N	Y	N	N	N	N	N	N	N										
112	untreated	Y	N	N	N	N	Y	N	Y	N	N										
115	untreated	N	Y	N	Y	N	N	N	N	N	Y										
121	untreated	N	Y	Y	N	N	Y	N	N	Y	N										
124	untreated	Y	Y	Y	N	N	Y	N	N	N	N										
137	untreated	Y	N	Y	N	N	N	Y	Y	Y	N										
155	untreated	Y	N	N	Y	N	Y	N	N	N	N										
156	untreated	Y	Y	N	N	N	Y	N	N	N	N										
158	untreated	N	Y	N	Y	Y	Y	N	N	Y	Y										
161	untreated	N	N	N	N	N	Y	N	N	N	N										
167	untreated	N	N	Y	Y	Y	Y	N	N	N	N										
170	untreated	N	N	N	Y	Y	N	N	N	N	Y										
175	untreated	Y	N	N	N	N	Y	Y	N	N	N										
192	untreated	Y	N	N	N	N	Y	N	Y	Y	N										
196	untreated	N	N	Y	N	N	N	N	N	Y	N										
202	untreated	Y	N	N	Y	N	Y	N	Y	Y	N										
203	untreated	Y	N	N	N	N	Y	N	N	N	Y										
208	untreated	N	N	N	N	N	N	Y	Y	N	N										
213	untreated	N	Y	Y	N	N	Y	N	N	N	Y										
218	untreated	Y	N	N	Y	N	Y	N	N	N	Y										
219	untreated	N	N	N	N	N	Y	N	N	N	N										
311	carprofen only	N	Y	N	N	Y	N	Y	N	N	Y										
312	carprofen only	Y	N	N	N	N	N	Y	N	N	N										
314	carprofen only	N	N	N	N	N	N	Y	Y	N	N										
315	carprofen only	Y	N	N	N	N	Y	N	N	Y	Y										
318	carprofen only	N	N	N	Y	N	Y	N	N	N	N										
319	carprofen only	Y	N	N	N	N	Y	N	N	N	Y										
322	carprofen only	Y	N	N	N	N	Y	N	N	N	Y										
323	carprofen only	Y	Y	N	N	N	N	Y	N	Y	N										
325	carprofen only	Y	N	N	N	N	Y	N	N	N	N										
327	carprofen only	Y	N	N	N	N	N	Y	N	N	N										
207	PBS	Y					Y			Y			Y	N	Y	N		Y	N		N
209	PBS					N	N				N	Y	Y	N	N			N	N	N	
215	PBS		Y		Y	N					N	Y			N		Y	Y	N	N	
216	PBS	Y					Y				N		Y	N	Y	N		Y	Y	N	
220	PBS			Y		Y	N	N			N	Y	Y		Y				N	N	
222	PBS			Y			N		N		N	Y	N		N	N		N		N	
231	PBS	Y		N			N	Y		Y			Y		Y	N			Y		Y
241	PBS	Y		Y			Y	Y		Y			Y		Y	Y			N		Y

242	PBS	Y		Y	Y		Y			Y			Y			Y		Y		Y	N
243	PBS	Y		N		N	Y			N	N		Y		N			N	Y		
280	50% EtOH[Contrast/PBS]	N	Y	Y			N				N				N	N		Y	N	N	
285	50% EtOH[Contrast/PBS]	Y	N	Y	N	Y	N	Y	N											N	N
286	50% EtOH[Contrast/PBS]	Y	N	N			Y			N	N				N	N		N	N		
287	50% EtOH[Contrast/PBS]	Y	N	N		N	N				N				N			N	N	Y	
288	50% EtOH[Contrast/PBS]	Y	N			Y	Y							N	N			Y	N	Y	N
290	50% EtOH[Contrast/PBS]	N	N		N	Y	N	N			N			N					N	N	
297	70% EtOH[Contrast/PBS]	Y	Y		Y		N				N			N		N		Y	Y	N	
304	70% EtOH[Contrast/PBS]	N	N	N		N	N		Y		N				N			N		N	
305	70% EtOH[Contrast/PBS]	Y				Y	N		Y		N		N	N	N			N		N	
308	70% EtOH[Contrast/PBS]	Y	N	N		N	Y			N	N				N			N	N		
309	70% EtOH[Contrast/PBS]	N	Y				N		Y	N				N	N	N		N			N
316	70% EtOH[Contrast/PBS]		Y			N	Y	N			N	N		N	N				N	N	
317	70% EtOH[Contrast/PBS]	Y			N	Y	Y		Y		Y		N	N				N		N	
324	70% EtOH[Contrast/PBS]	Y			Y	N	Y				N		N	Y				N	N	N	
338	70% EtOH[Contrast/PBS]			Y			Y				N	Y	N	N	N			N	N	N	
341	70% EtOH[Contrast/PBS]	Y					N		Y		N		N	N	N	N		N		N	
342	70% EtOH[Contrast/PBS]	Y		Y		N	Y		Y		Y		Y		N			N		N	
188	70% EtOH[H2O]	Y	N	N						N	N				N	N	N	N	N		
190	70% EtOH[H2O]	Y					N	N	N				N	N	N	N				N	N
193	70% EtOH[H2O]					N	Y	Y	N			N	N	N	N					N	N
200	70% EtOH[H2O]	N	Y	N			N	N	N						N	N				N	N
291	70% EtOH[H2O]		Y									N		N	N	Y	N	N	N	N	N
292	70% EtOH[H2O]	Y						Y	N	Y			N	N	N	N	N				N
294	70% EtOH[H2O]			Y								N	N		N	N	N	N	N	N	N
296	70% EtOH[H2O]							Y				N	N	N	N	N	N		N	N	N
299	70% EtOH[H2O]		Y									N		N	N	N	Y	N	N	N	N
300	70% EtOH[H2O]	Y			Y		Y						N	N		Y		N	N	N	N
301	70% EtOH[H2O]		Y						Y			N		N	N	N	Y	Y		N	N
307	70% EtOH[H2O]				Y							Y	N	N		N	N	N	N	N	N
310	70% EtOH[H2O]					Y		Y				N	N	N	N		Y		N	N	N

Supplementary Table 1. Tumor formation in non-injected and injected mammary glands assessed at necropsy. Presence of a tumor at necropsy is indicated with a “Y” for yes and absence with a “N” for no. Fields are left blank for mammary glands that were not part of that experimental group or class. Mammary gland location code: 1 = left cervical; 2 = left upper thoracic; 3 = left lower thoracic; 4 = left abdominal; 5 = left inguinal; 6 = right cervical; 7 = right upper thoracic; 8 = right lower thoracic; 9 = right abdominal; 10 = right inguinal.
